# Predictors of mortality among under-five children with severe acute malnutrition, Northwest Ethiopia: an institution based retrospective cohort study

**DOI:** 10.1186/s13690-018-0309-x

**Published:** 2018-09-27

**Authors:** Fasil Wagnew, Debrework Tesgera, Mengistu Mekonnen, Amanuel Alemu Abajobir

**Affiliations:** 1 College of Health Sciences, Debre Markos University, Debre Markos, Ethiopia; 2 College of Health Sciences, University of Gondar, Gondar, Ethiopia; 3 Faculty of Medicine, The University of Queensland, Brisbane, Australia

**Keywords:** Mortality, Severe acute malnutrition, Ethiopia

## Abstract

**Background:**

Globally, approximately 19 million children under 5 years are suffering from Severe Acute Malnutrition (SAM). It is a major cause of morbidity and mortality in low-income countries including Ethiopia. However, little is known regarding predictors of mortality among these children in Ethiopia. The current study aimed to assess the potential predictors of mortality among under-five children with SAM admitted to a stabilization center.

**Method:**

A retrospective cohort study was conducted in 527 under-five children who were admitted for SAM at the University of Gondar comprehensive specialized hospital from 2014 to 2016. Data were collected from a randomly selected chart after getting ethical clearance. Data were cleaned, coded and entered to Epi-info (version 7) and analyzed using STATA (version14). The outcome was computed by using tables and graphs. A multivariable cox proportional hazards model was fitted to identify predictors of mortality.

**Result:**

Overall, the median follow-up period was 10 days with interquartile range (Q1, Q3: 8, 17). At the end of the follow-up, the mortality rate was 66(12.52%). Anemia (AHR(Adjusted Hazard Ratio): 2.3, 95% CI: 1.2, 4.5), Shock (AHR: 7.9, 95% CI: 3.7, 16.7), no intake of antibiotics (AHR: 2.3 95% CI: 1.2, 4.4), IV-Fluid (AHR: 3.2, 95% CI: 1.7, 5.8), no intake of F75 (AHR: 6.6,95% CI: 2.9, 14.7) and no intake of F100 (AHR: 3, 95% CI: 1.6, 5.4) were independent predictors of mortality.

**Conclusion:**

The survival status of under-five children with SAM was lower than the national standard protocol. Altered general conditions such as shock, anemia, not adhering to medical and nutritional therapies were identified as predictors of mortality among SAM children. Health education on early medical seeking behavior and adherence on the routine regimens may improve this gap in child survival.

**Electronic supplementary material:**

The online version of this article (10.1186/s13690-018-0309-x) contains supplementary material, which is available to authorized users.

## Background

Malnutrition involves both under-nutrition and over-consumption, causing severe outcomes on human body structure and function with specific physical and clinical consequences [1].

Childhood under-nutrition incorporates combination of nutritional disorders that include underweight, wasting, stunting and micronutrient deficiency [2]. Underweight, based on weight for-age, is a composite measure of wasting and stunting. Wasting (weight for height) is an acute malnutrition due to a recent failure to receive adequate nutrition and may be affected by recent episodes of diarrhea and other acute illnesses [3]. By contrast, stunting (chronic malnutrition) is a measure of growth retardation showing the cumulative effect of chronic food deprivation [4]. Acute malnutrition classified as moderate acute malnutrition (MAM) and Severe Acute Malnutrition (SAM) based on their severity [5]. SAM is defined as weight for height below − 3 z scores of the median WHO growth standards or presence of bilateral edema or Mid Upper Arm Circumference (MUAC) < 115 mm for a child ≥ 6 months age [6].

SAM remains one of the major public health problem and an important contributor to child morbidity and mortality in the world [1]. Globally, approximately 19 million children under 5 years suffered from SAM in 2015. Children with SAM are nine times more likely to die as compared to healthy children [7, 8]. Mortality rates as low as 2.2% in India [5] and as high as 42% in Malawi [6] were observed among children treated in stabilization center. Likely causes of this high inpatient mortality are inappropriate case management [9], SAM with co-morbidities like diarrhea, pneumonia, malaria, and tuberculosis, as well as poor compliance to different types of medical and nutritional therapeutic alternatives [10].

A vast majority (over 90%) of SAM is located in South and Southeast Asia and sub-Saharan Africa [11] and is a common indication for hospital admission and treatment among pediatric patients. Limited inpatient capacity, and inadequate trained staff available in hospitals to treat the large numbers requiring care, has long been known to limit impact and programme coverage [12, 13]. Lately, community-based therapeutic care programme treating most cases of SAM solely as outpatients have dramatically reduced case fatality rate, particularly in Bangladesh, South Sudan, Angola, Ethiopia, and Malawi [14–16].

In Ethiopia, SAM is a major public health and economic problem with increased cost institution, family and indirect cost [17] and it is the preliminary diagnosis in 20% of pediatric hospital admissions [18]. The Ethiopian demographic health survey (EDHS) reported that overall, 10% of children in Ethiopia are wasted, of which 3% are severely wasted. Regional variations exist, with Somali and Afar having the highest percentages of children who are wasted, 23% and 18%, respectively [19]. According to Health and Health Related Indicators (HHRI) 2014, in Ethiopia; SAM was the third leading cause of mortality and accounted for 8.1% of the deaths of under-five children [20]. A report from Dollo Ado district Somali, states that 42.3% of children were acutely malnourished with 16.3% was severely wasted [21]. Considering this high trouble of under nutrition, the Ethiopian government launched “Seqota declaration” with nutrition as one of the nutritional agenda to end under nutrition by 2030 [22]. Despite the availability of treatment for children with SAM in stabilization centers, the fatality rates for inpatient management of SAM still remain high in Ethiopia. Therefore, this study aimed to determine predictors of mortality in SAM children admitted to University of Gondar comprehensive specialized hospital (UOGCSH).

## Methods

### Study design, period and population

An institutional based retrospective cohort study was used by reviewing medical records from March–April, 2017. The hospital is used as a referral center for North Gondar administrative district and other catchment area. It has 512 bed capacities. Pediatrics ward has a separate room used as a treatment center for SAM children. Health personnel follow an updated and a standardized form of treatment protocol of SAM guideline [23]. According to this protocol, all SAM cases with medical complication and poor appetite are admitted to the hospital for inpatient management. The source population was all under-five children with SAM admitted to stabilization centers at the UOGCSH. The study population included randomly selected eligible under-five children with SAM admitted to therapeutic feeding unit (TFU) at the UOGCSH, from January1/2014 to December 30/2016.

### Inclusion criteria

Full records of all under-five children with SAM admitted to TFU at UOGCSH, between January 1/2014 to December 30/2016 were recruited.

### Exclusion criteria

Children with incomplete records were excluded from the study.

### Sample size and sampling technique

The sample size was computed by using STATA (version 14) by the following statistical assumptions considered, two-sided significant level (α) of 5%, power 80%, Z_a/2_ = Z value at 95% confidence interval = 1.96, death rate = 29%, Hazard Ratio (HR) = 1.53 [10]. A total of 1223 children with SAM were admitted to this hospital from January1/2014 to December 30/2016. Open-Epi software (version 3) was used to generate a random sample. Then, using the subsequent unique SAM number or from SAM registry, a random sample of 570 children was selected.

### Data collection procedure

A checklist was built up from the standard treatment protocol for the management of SAM, SAM registration booklet, SAM multi-chart and reviewing applicable articles to assemble the required individual information. The data extraction format consists of the patient related data (age, sex, residence), anthropometric measurements, (height, weight, MUAC, edema), co-morbidities, types of SAM (marasmus, kwashiorkor or marasmic-kwashiorkor), feeding phase and types of feeding (F75 or F100), frequency of feeding and amount per feed, as well as medication given and outcomes of the treatment. The data collection checklist was organized by using the standardized entry based on regular data registration protocol. The data extraction tool was checked for the completeness and consistency using 5% pre-tested randomly selected charts. Three professional data collectors and one supervisor were recruited, who had training and experience on SAM management. They also received a two day training to standardize and agree on the way to review medical records (Additional file 1).

### Data processing and analysis

Data were entered and cleaned by Epi-info (version 7), and exported to STATA (version 14) for further analysis. Exploratory data analysis was carried out to check the levels of missing values, possible outliers, and multicollinearity. The weight, height, and edema were further exported to WHO anthro software (2010) to calculate a WFH Z score at admission. To identify predictors associated with death rate, Cox proportional hazard model with hazard ratio of 95% CI was used. Variables at *p* < 0.25 level in the bi-variable analyses and stepwise forward variable selection was computed so as to identify eligible variables in the final cox-regression model to identify independent predictors of mortality. All statistical tests were considered significant at 0.05 or 5%. The Cox regression model for its fitness to the data and proportional hazard assumptions was checked by using both log-log plot and Schoenfeld residuals test. Model comparisons were also computed by using log-likely hood ratio test and Harrell’s concordance statistics test. Furthermore, unsteadiness of parameter estimate among variables in the final fitted model was checked by using Variance Inflation Factor (VIF) and goodness of fit of the final model was checked by Nelson Aalen cumulative hazard function against cox-Snell residual.

### Operational definitions

**Censoring**: right censoring, are those cases as defaulters, recovered or none recovered.

**Event:** Death.

**Defaulter**: Patient that is absent for 2 consecutive days.

**Non-recovery:** Patient that has not reached the discharge criteria after 40 days in the inpatient program.

**SAM:** defined as weight for height below − 3 z scores of the median WHO growth standards or presence of bilateral edema or mid upper arm circumference < 115 mm for a child ≥ 6 months age.

**Survival:** No experience of death during hospitalization period or it is being alive and not experiencing SAM related death during hospitalization period.

**Length of stay:** The number of days the child stayed in the hospital from admission until death or censoring.

## Results

### Socio-demographic and admission characteristics

A cohort of 527 SAM children was followed for a median time of ten days with an inter-quartile range (Q1, Q3: 8, 17). From this 277 (52.56%) were male and about three-quarters (75.14%) came from rural areas. The mean age was 19 months with a standard deviation of 15.6 months. About half (50.3%) were between one and thirteen months. Most children 349 (66.2%) suffered from non-edematous types of malnutrition (Table 1).

**Table 1:** Sociodemographic characteristics of children with SAM admitted in UOGCSH from 2014 to 2017, Gondar, Northwest Ethiopia (*n* = 527)

Characteristics	Frequency	Percent (%)
Ag		
≤ 24 months	418	79.32
> 24 months	109	20.68
Sex		
Male	277	52.56
Female	250	47.44
Residence		
Urban	131	24.86
Rural	396	75.14
Appetite test		
passed appetite	159	`49.69
Failed appetite	161	50.31
Nutritional edema		
Yes	178	33.78
No	349	66.22
MUAC		
≤ 11.5 cm	331	75.7
> 11.5 cm	106	24.3
WFH		
below z-score − 3	252	50.5
Z score ≥ −3	247	49.5
History of bottle feeding		
yes	124	23.98
No	393	76.02

*MUAC* Mid Upper Arm Circumference

*WFH* Weight For Height

As shown in (Table 2) concerning to the presence of co-morbidities with SAM at admission, diarrhea and pneumonia were recorded as frequent co-morbidities among admitted SAM children. Accordingly, about 204 (38.71%) and 74 (14.04%) children had diarrhea and pneumonia as major co-morbidity conditions.

**Table 2:** Foremost medical co-morbidities among children with SAM in UOGCSH from 2014 to 2017, Gondar, Northwest Ethiopia (*n* = 527)

Characteristics	Frequency	Percent
HIV/AIDS		
Reactive	18	3.42
Non-reactive	332	63.00
Unknown	177	33.59
Tuberculosis		
Yes	51	9.68
No	476	90.32
Pneumonia		
Yes	74	14.04
No	453	85.96
Diarrhoea		
Yes	204	38.71
No	323	61.29
Malaria		
Yes	14	2.66
No	513	97.34
Others		
Yes	26	4.93
No	501	95.07

*HIV/AIDS* Human Immune-Virus /Acquired Immune Deficiency Syndrome

### Mortality rate of children with SAM and their survival status

**Fig. 1:**
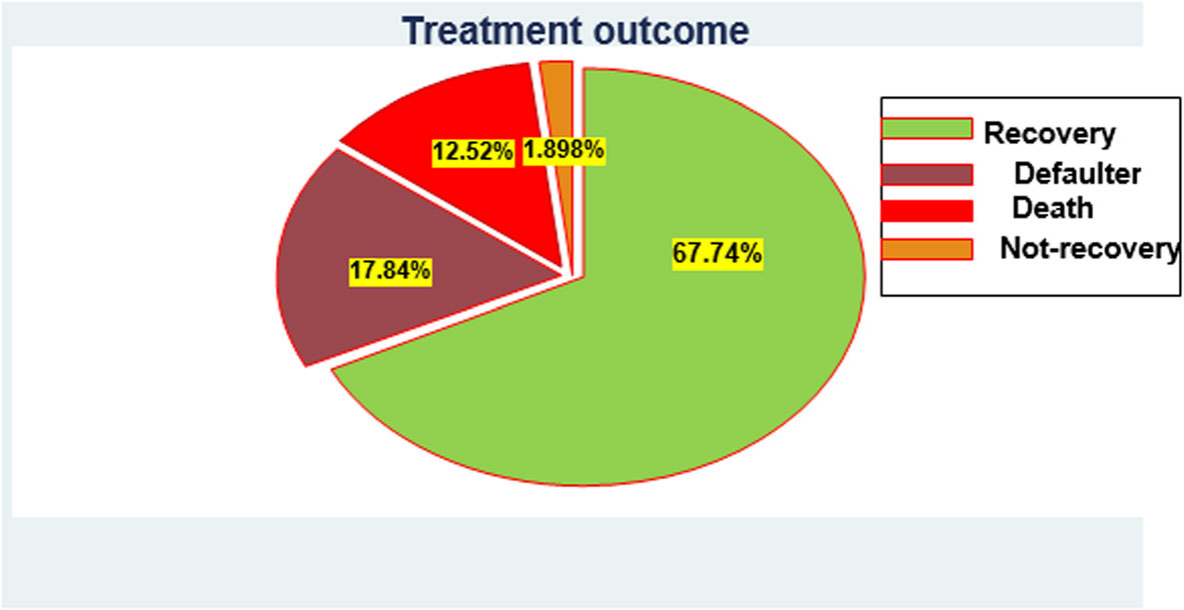
Treatment outcome of inpatient SAM children in UOGCSH from 2014 to 2017, Gondar, Northwest Ethiopia

Regarding treatment outcomes of SAM, 66 (12.52%) children were dead and 357 (67.7%) were recovered at the end of follow-up (Fig. 1). The total time at risk for 527 SAM children was 6333 days with an incidence rate of 10.4 deaths /1000 child-days. The average length of stay in the hospital was 12 days, whereas the average rate of weight gain was 29.1 g/kg/day, with a lower rate of 4.0 g/kg/day and a higher rate of 40 g/kg/day was depicted for edematous and non-edematous.

**Fig. 2:**
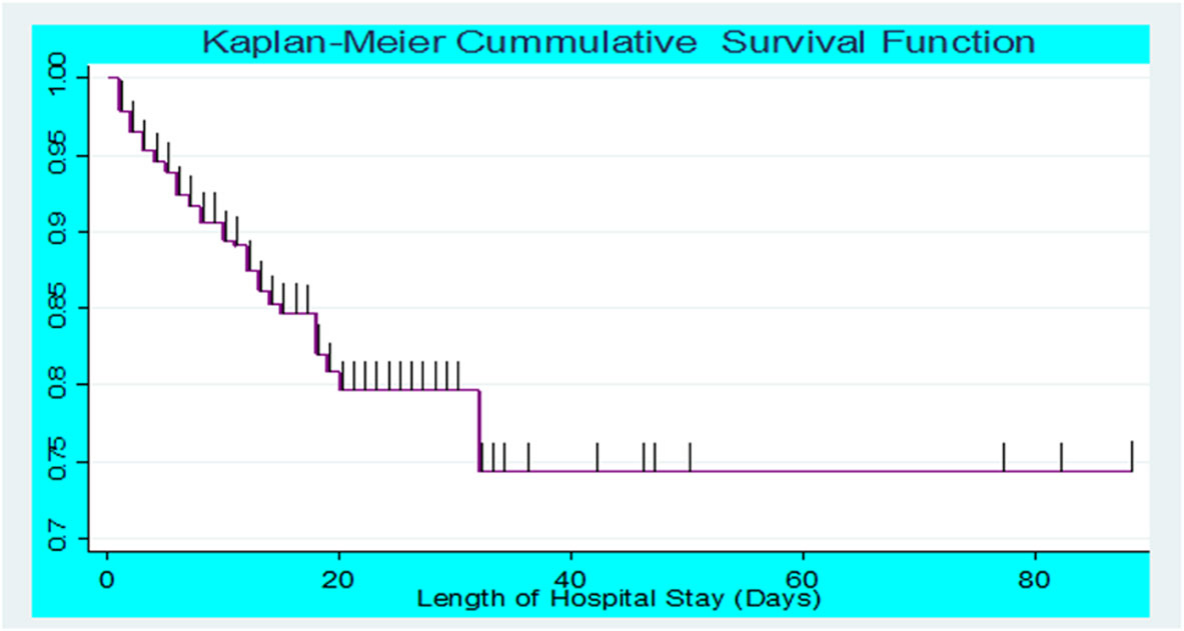
Overall Kaplan-Meier estimation of their survival of admitted SAM children in UOGCSH from 2014 to 2017, Gondar, Northwest Ethiopia

As shown in Fig. 2, most of the death occurs during the first few days of admission to the hospital. That is, 59 deaths occurred within the first couple of weeks of admission to inpatient SAM treatment center. The cumulative probability of survival at the 5th, 10th and 15th day was 90.2%, 84.7%, and 80.9%, respectively, whereas the overall mean survival time for this study was 69 days (95% CI:62.3–76).

### Predictors of mortality among SAM children

From the bi-variable analyses, anemia, shock, NGT (Naso-Gastric Therapy), no intake of antibiotics, Iv-fluid, altered pulse rate at admission, no intake of F75 and F100 were significant predictors of mortality in SAM children (see more Appendix).

After adjusting for other variables, children with anemia had more than two times hazard of death as compared to children without anemia (AHR: 2.3,95% CI: 1.2, 4.5). Risk of earlier death for children with shock was nearly eight times higher than for children without shock (AHR: 7.9, 95% CI: 3.7, 16.7). And also, children who did not take routine antibiotics were about two times hazard of death as compared to those managed by routine antibiotics (AHR: 2.3,95% CI: 1.2, 4.4). Likewise, the hazard of mortality was more than three times higher among children who required IV-fluid than their counterparts (AHR: 3.2, 95% CI: 1.7, 5.8) (Table 3).

**Table 3:** Bi-variable and multivariable Cox- regression analysis for independent predictors of mortality among under-five children with SAM admitted in UOGCSH from 2014 to 2017, Gondar, Northwest Ethiopia(*n* = 527)

Variables	Event	Censored	CHR, 95% CI	AHR and 95% CI	*p*-value
Diarrhoea					
Yes	31	173	1.66 (0.8–2.69)	1.37 (0.79–2.4)	0.254
No	35	288	1	1	
Heart failure					
Yes	65	458	1.39 (0.63–3.06)	2.35 (0.91–6.02)	0.07
No	58	427	1	1	
Hiv/Aids					
Reactive	6	12	6.84 (0.43–19.2)	1.5 (0.45–4.97)	0.507
Non-react	51	281	2.96 (0.46–6.02)	1.6 (0 .75–3.4)	0.215
Unknown	9	168	1	1	
Pneumonia					
Yes	9	65	0.93 (0.46–1.88)	1.1 (0.5–2.45)	0.8
No	57	396	1	1	
Folic acid					
Yes	36	346	1	1	0.525
No	30	114	2.4 (1.48–3.9)	1.2 (0.67–2.16)	
Anemia(< 11 mg/dl)					
Yes	49	221	2.57 (1.46–4.53)	2.37 (1.24–4.51)	0.023*
No	16	226	1	1	
Shock					
Yes	34	9	19.02 (11.65–31)	7.95 (3.72–16.7)	< 0.001*
No	32	449	1	1	
NGT feeding					
Yes	44	154	3.35 (2.0–5.61)	1.56 (0.7–2.04)	0.1
No	22	307	1	1	
Routine Antibiotics					
Yes	24	272	1	1	0.007*
No	42	189	2.46 (1.49–4.07)	2.35 (1.25–4.4)	
Iv fluid					
Yes	48	77	9.61 (5.58–16.5)	3.21 (1.75–5.88)	< 0.001*
No	18	384	1	1	
Blood transfusion					
Yes	27	31	6.36 (3.8–10.4)	1.9 (0.93–3.88)	0.077
No	39	430	1	1	
Intake of F75					
Yes	51	377	1	1	< 0.001*
No	15	84	1.70 (0.95–3.04)	6.58 (2.9–14.68)	
Intake F100					
Yes	24	315	1	1	< 0.001*
No	42	146	5.27 (3.15–8.79)	3 (1.67–5.41)	
Deworming					
Yes	5	54	1	1	0.45
No	61	407	1.8 (0.9–9.2)	1.61 (0.46–5.64)	
Pulse					
Normal	20	243	1	1	0.37
Altered	46	211	2.34 (0.39–3.97)	2.39 (0.24–4.61)	

*Significant predictors in the multivariable analysis

*HIV/AIDS* Human Immune-Virus /Acquired Immune Deficiency Syndrome

*NGT* Naso Gastric Therapy

## Discussion

The present study determines the predictors of mortality among under-five children with SAM. The treatment outcomes of the current study shows a death rate of 12.5%, a cure rate of 67.74%, a defaulter rate of 17.84% and a non-recovery rate of 1.9%. The proportion of children who died (12.52%) was higher than the minimum sphere standard and national management protocol for SAM as well (< 10%) [24]. This high death rate could be a result of delay presentation to the stabilization center, remarkably on the first day of admission, and also parents might discontinued children’s treatment courses due to financial limits to buy drugs and foods.

This death rate was comparable with a study done in Mekele (12.8%) [25], but finding was higher than in the study done in Malawi (7.7%) [26], in Ethiopia Woldiya Hospital 21(6%) [27], in Jimma University Specialized Hospital 88 (9.3%) [28], Dilchora Referral Hospital 7.6% [29]. On the other hand, this finding is lower than the study done in Sekota Hospital 29% [10]. The potential elucidation for the disparity could be that caseload, proportion of co-morbidity and severity of cases were lower in our study area.

The average length of stay in the hospital for 12 days with 29.1 g/kg/day average rate weight gain was in line with the minimum sphere standard average length of wait that should not exceed 30 days [24]. This could be due to the occurrence of recurrent infections, presence of co-morbidity, and socioeconomic status. Though, the overall average length of stay in the hospital was longer than in other studies [29, 30]. This might be because of the harshness of medical conditions of children in this particular population. In sum, the present study revealed that the mean survival time of 69 days was consistent with the study done in Dilchora Referral Hospital also reported a mean survival time of 69 days [29]. This finding is also supported by another study in Gedeo zone [31], which found that the mean survival time was 79.6 (95% CI: 67.5, 91.8) days. Furthermore, as indicated in the result of this study, the cumulative probability of survival at 5th, 10th and 15th day was 90.2%, 84.7%, and 80.9%, respectively with a difference between groups of variables.

Regarding the predictors, SAM patients with anemia had more likely hazard of death as compared to children without anemia. The risk of earlier death was higher for children with shock than children without shock. This was in accordance with the finding of Gebremichael [25] which demonstrated that anemic and shock children were more likely hazard of death as compared to their counterparts. This finding also held by another study done by Desta [10] which revealed that the hazard rate of death among children with severe anemia was higher than children with no anemia. This is due to the fact that there is an increased risk of infection among anemic children and also children with SAM who present with shock have a drastically high risk of death [8]. Even though the current study did not show any significant association between children with tuberculosis, pneumonia, HIV/AIDS and mortality, other studies were done in Zambia [32] and Ethiopia [10, 28, 29] indicated that SAM children with major co-morbidities were at higher risk of mortality. This controversy might be due to sticky to current WHO and national SAM treatment guideline and early detection as well as giving prophylaxis.

Accordingly, the hazard of mortality was higher among children who required IV-fluid than their counterparts. This finding consistent with the previous study [25], and could be due to the fact that the use of IV-fluid might lead to secondary complications including fluid overload, cerebral edema, heart failure and infection. These could contribute to death for children with SAM due to loss of intracellular potassium in the extracellular space and decrement of total body potassium. The overall adaptive responses to repeated infections and subsequent adaptive physiological changes such as reduced renal and cardiac output may increase proneness to infection [8]. Likewise, non-adherence to nutritional therapies such as F75 and F100 were significant predictors of mortality for SAM children [29] possibly due to metabolic derangements [8]. In other words, appropriate nutritional therapy based on the national SAM management protocol promotes early recovery and decreases the risk of death. Those children did not adhere to routine antibiotics had the higher hazard of death as compared to those children took routine antibiotics properly. This is consistent with randomized, double-blind, placebo-controlled trial study done in Malawi reported that the proportion of children who received placebo has a significantly lower recovery as compared to children who received antibiotics [33]. This might be because of the heightened risk of severe bacterial infections on account of decreased immunity in such children. In general, screening, case identification, treatment, referral and follow up of cases of children with SAM at the community level should be given due emphasis. These may be the most effective and efficient way to mitigate complicated SAM and consequent mortality at hospital level. Decision makers or other concerned body should monitor and evaluate therapeutic feeding programs to reduce mortality and give emphasis on medication and nutrition adherence for cases with complication including anemia and shock.

### Limitation of the study

This study has some important limitations that should be considered cautiously while interpreting the results. The data were retrospectively extracted from patients’ medical records, and some relevant variables such as child’s immunization status, additional family meal, and appetite test were inadequately recorded and were not included in the analysis. Potential bias related with excluded records and the health workers who treated patients were of different educational background (pediatricians, general practitioners, public health and nurses) and those who did the measurements had also of different levels of attitude and experience. Knowing of the fact that these compromise the quality of the reports, standard training and regular supervision.

To complement the limitations of this study, further study using prospective study design would better compensate these limitations.

## Conclusion

The overall survival status of under-five children with SAM was lower than the national standard protocol. Altered general conditions such as shock, anemia, not adhering to medical and nutritional therapies were identified as predictors of mortality among SAM children. Health education on early medical seeking behavior and adherence on the routine regimens may improve child survival.

## Additional file

Additional file 1: Data abstraction tool. (DOCX 26 kb)

## Appendix

**Table 4:** Bi-variable Cox- regression analysis for independent predictors of mortality among under-five children with SAM admitted in UOGCSH from 2014 to 2017, Gondar, Northwest Ethiopia, May 2017 (*n* = 527)

Variables		Outcome		CHR and 95% CI	*p*-value
		Event	Censored		
Age category	≤ 24 months	51	367	1.04(.61–1.94)	0.7
	> 24 months	15	94	1	
Sex	Male	31	246	1	
	female	35	215	1.27 (0.78–2.06)	0.33
Residence	Urban	15	116	1	
	Rural	51	345	1.01 (0.56–1.8)	0.968
SAM type	Marasmic	36	313	1	
	kwashiorkor	10	106	0.79 (0.39–1.59)	0.51
	Marasmic kwashiorkor	20	42	2.3 (0.5–3.59)	0.43
TB	Yes	5	46	0.67 (0.27–1.68)	0.39
	No	61	415	1	
HIV test	Reactive	6	12	6.84 (0.43–19.22)	0.16
	Non-reactive	51	281	2.96 (0.46–6.02)	0.13
	Un-known	9	168	1	
Malaria	Yes	1	13	0.70 (0.09–5.09)	0.72
	No	65	448	1	
Diarrhoea	Yes	31	173	1.66 (0.8–2.69)	0.09
	No	35	288	1	
Dehydration	Yes	22	92	1.43 (0.12–3.57)	0.28
	No	44	369	1	
Anemia(< 11 mg/dl)	Yes	49	221	2.57 (1.46–4.53)	0.001*
	No	16	226	1	
kuashdermatitis	Yes	8	42	1.2 (0.10–1.61)	0.31
	No	58	419	1	
Shock	Yes	34	9	19.02 (11.65–31.06)	0.000*
	No	32	449	1	
Pneumonia	Yes	9	65	0.93 (0.46–1.88)	0.242
	No	57	396	1	
Heart failure	Yes	65	458	1.39 (0.63–3.06)	0.14
	No	58	427		
NGT insertion	Yes	44	154	3.35 (2.0–5.61)	0.000*
	No	22	307	1	
Vitamin A	Yes	36	319	1	
	No	30	142	1.69 (0.65–3.38)	0.3
Folic acid	Yes	37	346	1	
	No	29	114	1.4 (0.48–3.9)	0.29
Ant malaria	Yes	2	18	1	
	No	64	443	1.44 (0.35–5.93)	0.6
Deworming	Yes	5	54	1	
	No	61	407	2.88 (.90–9.19)	0.07
Antibiotics	Yes	24	272	1	
	No	42	189	2.46 (1.49–4.07)	0.000*
Resomal	Yes	46	279	1.37 (0.81–2.32)	0.36
	No	20	182	1	
Iv fluid	Yes	48	77	9.61 (5.58–16.5)	0.000*
	No	18	384	1	
Blood transfusion	Yes	27	31	6.36 (0.8–10.4)	0.21
	No	39	430	1	
Pale-conjunctiva	Yes	19	63	2.22 (0.30–3.80)	0.33
	No	47	398	1	
Intake of F75	Yes	51	377	1	
	No	15	84	1.70 (0.95–3.04)	0.07*
Intake F100	Yes	24	315	1	
	No	42	146	5.27 (3.15–8.79)	0.000*
Temperature	Normal	46	416	1	
	Altered	20	45	2.85 (0.34–6.32)	0.39
Pulse	Normal	20	243	1	
	altered	46	211	2.34 (1.39–3.97)	0.001*
Respiration	Normal	27	263	1	
	altered	39	192	1.89 (0.15–3.08)	0.72

## Abbreviations

AHR: Adjusted Hazard Ratio
CHR: Crude Hazard Ration
HIV/AIDS: Human Immune-Virus /Acquired Immune Deficiency Syndrome
MUAC: Mid-Upper-Arm-Circumference
NGT: Naso-Gastric Tube
SAM: Severe Acute Malnutrition
UOGCSH: University of Gondar Comprehensive Specialized Hospital
WFH: Weight for Height
WHO: World Health Organization
